# Explainability of the COVID-19 epidemiological model with nonnegative tensor factorization

**DOI:** 10.1007/s41060-022-00324-1

**Published:** 2022-04-30

**Authors:** Thirunavukarasu Balasubramaniam, David J. Warne, Richi Nayak, Kerrie Mengersen

**Affiliations:** 1 School of Computer Science, Queensland University of Technology, 2 George Street, Brisbane, QLD 4000 Australia; 2 Centre for Data Science, Queensland University of Technology, 2 George Street, Brisbane, QLD 4000 Australia; 3 School of Mathematical Sciences, Queensland University of Technology, 2 George Street, Brisbane, QLD 4000 Australia

**Keywords:** COVID-19, Stochastic epidemiological modelling, Explainable AI, Nonnegative tensor factorization, Clustering, Pattern mining

## Abstract

The world is witnessing the devastating effects of the COVID-19 pandemic. Each country responded to contain the spread of the virus in the early stages through diverse response measures. Interpreting these responses and their patterns globally is essential to inform future responses to COVID-19 variants and future pandemics. A stochastic epidemiological model (SEM) is a well-established mathematical tool that helps to analyse the spread of infectious diseases through communities and the effects of various response measures. However, interpreting the outcome of these models is complex and often requires manual effort. In this paper, we propose a novel method to provide the explainability of an epidemiological model. We represent the output of SEM as a tensor model. We then apply nonnegative tensor factorization (NTF) to identify patterns of global response behaviours of countries and cluster the countries based on these patterns. We interpret the patterns and clusters to understand the global response behaviour of countries in the early stages of the pandemic. Our experimental results demonstrate the advantage of clustering using NTF and provide useful insights into the characteristics of country clusters.

## Introduction

While the world has witnessed many infectious diseases in the past, the current COVID-19 pandemic occurs in the era of data and computing advancements. We have access to a vast amount of historical data from which we can learn and apply the knowledge gained to tackle the current COVID-19 pandemic. Additionally, a large amount of real-time COVID-19-related data is being generated and shared globally seamlessly [12]. Modelling such datasets and understanding their outcomes can assist government and policymakers to make informed decisions [7].

Stochastic epidemiological models (SEMs) are widely used to simulate the spread of an infectious disease based on the reported case data [3]. These models can quantify the uncertainties in parameters like transmission rate, response rate, recovery rate, and many others, which in turn, allow simulating the spread of the infectious disease and the range of possible outcomes. Several studies have simulated the spread of COVID-19 based on the parameters learned from historical and similar diseases [8, 11, 16]. Simulation studies and forecasts of COVID-19 spread have been crucial in containing the spread through non-pharmaceutical interventions (NPI) [24, 29].

Over two years into the COVID-19 pandemic, the COVID-19 virus is mutating into many new variants [17]. Each new variant seems to be more dangerous than the initial COVID-19 strain, and hence poses more challenges in understanding and tackling them including vaccine effectiveness [25]. While the past research has focused on the simulation of COVID-19 spread, it is crucial now to focus on the explainability of those models for knowledge discovery. This will help us to learn what has worked and what has not worked in containing the spread of COVID-19 in different countries.

Warne et al. [34] investigated the response behaviour of 158 countries using an SEM that includes changes in the community spread in response to reported cases. The inferred parameters related not only to the virus transmission, but also the response behaviour of each country leading to different outcomes of COVID-19 spread. They employed a Bayesian inference approach to infer the regions based on the reported confirmed cases, recoveries, and fatalities. This Bayesian inference model includes more detailed information; however, its interpretation (i.e. explainability) to effectively draw insight about the disease dynamics is difficult. This model generates samples (particles) from the joint parameter posterior distributions for each country analysed. The output, i.e. parameter posterior distribution, is three-dimensional (i.e. *parameters* inferred from *particles* for *countries*) which needs special attention for explainability.

In this paper, we apply nonnegative tensor factorization (NTF) in a novel fashion to represent and learn the parameter patterns of an SEM. SEM learns the latent epidemiological parameters based on the infectious disease cases and the NTF model reveals latent features of countries based on the latent epidemiological parameters learned using SEM. In other words, NTF is applied to decode the latent epidemiological parameters learned from SEM and gain more insights. More specifically, the contributions of this paper are twofold:

1. We represent the output of the epidemiological model as a tensor model.
2. We demonstrate the utility of NTF to identify patterns in global response parameters and provide explainability of the epidemiological model based on the work of Warne et al. [34].

An advantage of the proposed approach is that it is generic and can be applied to understand the global response parameters’ patterns of any infectious disease, given an appropriate model including response dynamics is available.

## Related works

A susceptible infectious recovered (SIR) model [18] is a fundamental epidemiological model that is widely applied to understand the likely infectious disease spread based on the known number of susceptible, infectious, and recovered cases in a population. Cooper et al. [11] simulated the spread of COVID-19 using an SIR model to analyse the dataset collected from January to June 2020 by countries including China, South Korea, India, Australia, USA, Italy, and Texas State (USA). Since only three compartments (i.e. susceptible, infectious, recovered) are used in the model, the simulation of COVID-19 spread will have limitations to a more general understanding. Depending on the availability of COVID-19 data, many variants of SEMs are applied. Carcione et al. [8] simulate the COVID-19 spread using an SEIR model that includes an additional latent infectious component of Exposed (E). Similarly, authors in [16] simulate the spread of COVID-19 in Italy by introducing additional compartments such as diagnosed, ailing, recognized, healed, and extinct. These models include many compartments and make the simulation of COVID-19 spread enriched with details. The main focus of these models is to simulate the spread of COVID-19 for future forecasting. Warne et al. [34] applied Bayesian methods to analyse the reported case data to infer key epidemiological parameters along with 95% credible regions, that is, the region containing 95% of the posterior probability. This model includes more detailed information; however, its interpretation for effectively drawing insight about the disease dynamics is difficult. While future forecasting is good, it is equally important to understand how each country employs the response strategies. This insight will reveal the factors that assist in combating a disease.

Advancements in machine learning techniques and the availability of data have attracted extensive research on the development of machine learning techniques to understand more about COVID-19 [2, 4, 23]. For example, Kushwaha et al. [23] study various machine learning algorithms to learn useful characteristics of COVID-19 to better understand the drugs development. Similarly, learning the characteristics of COVID-19 in any form is advantageous to effectively fight the disease. Balasubramaniam et al. [4] applied a machine learning algorithm, especially a clustering algorithm, on text data generated by social media posts to understand the topic dynamics and their spatiotemporal spread. Such outcomes can be used to understand how people react to COVID-19.

Clustering is a popular descriptive mining technique used that groups the observations based on characteristic similarities. It provides explainability to the data by providing common characteristics of each group. If the point estimates of parameters inferred from the epidemiological model for each country are known, clustering can be applied to group the countries based on similar parameters. The characteristics of each cluster can be defined by the common parameters and their values. However, the output of the detailed Bayesian analysis of the SIR models [34] is a set of samples from the parameter posterior distribution that is represented by three-dimensional values of estimates for parameters at different samples for different countries.

In this paper, clustering applied on the parameters inferred from the epidemiological model is an attempt to improve the explainability of the epidemiological model. While clustering, in general, makes the epidemiological model explainable, the incorporation of the posterior distribution (inferred from different particles in the Bayesian inference model) in clustering is challenging. The standard algorithms such as KMeans [20], DBScan [14], and nonnegative matrix factorization (NMF) [27] will fail due to their requirement of a two-dimensional matrix data structure. The output of the Bayesian inference model, i.e. parameter posterior distribution is three-dimensional (parameters inferred from particles for countries) which needs a third-order tensor model for representation. NTF, an extension of NMF for higher order, can utilize a three-dimensional data and can cluster the observations. Additionally, the outputs produced by NTF give more interpretable outputs. For example, Balasubramaniam et al. [4] used NTF to cluster COVID-19-related tweets and used the output to interpret clusters as topics along with their spatiotemporal patterns.

In this paper, we propose to apply NTF to cluster the countries based on parameters and to understand the patterns of parameters. The usage of NTF or any factorization-based methods to improve the explainability of parameters inferred in a SIR model is not studied before and this work will be the first of its kind. Recently, NTF is utilized in STELAR [21] where the SIR model is used as an integral component (i.e. regularization) in the factorization framework for forecasting the transmission of the infectious disease. Different from this work, the goal of our research is not to predict but to understand/characterize the countries based on the SIR model parameters using NTF.

## SEM to Infer Community Responses

SEMs have been extensively used to understand the spread of COVID-19 [8, 11] as well as to inform and assess response measures [24, 29, 34]. An SIR model is a popular SEM that simulates the spread of infectious disease in a population based on three states (also called compartments): (1) susceptible (*S*), (2) infectious *I*, and (3) recovered *R*. *S* represents the number of people in a population who have no immunity against COVID-19 and are at risk of being infected with the disease. *I* measures the number of people infected in the population, whereas *R* is the number of people that can no longer spread infection due to either recovering (and obtaining immunity) or dying. For any given population, people will belong to either of these states. SIR models learn latent variables as the model will use parameters such as contact and recovery rates to learn the SIR states that describe the spread of the disease. The true distribution of a population is only partially observable based on the reported case data. For example, the Johns Hopkins University (JHU) dataset [12] provides public data on COVID-19.

**Fig. 1:**
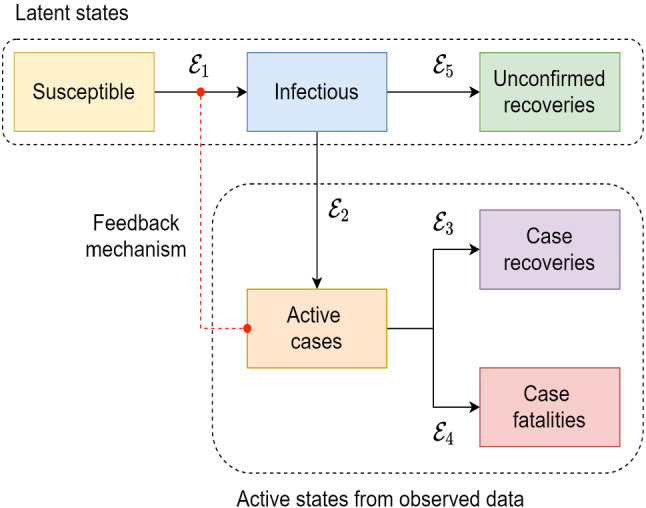
Overall architecture of the SEM with multiple components [34]. A feedback mechanism also incorporates the community influence in the process

There exist many variations of SIR models to incorporate additional states/compartments. Warne et al. [34] developed a new compartmental model based on the JHU dataset [12] and simulated the spread of COVID-19 using six compartments. The JHU dataset consists of each countries’ active cases (*A*), recovered cases (*R*), and death cases (*D*). Since *S*, *I*, and *Ru*^1^ are not observable in the dataset, the strategies for containing the spread of COVID-19 are made based on the observed *A*, *R*, and *D* compartments. Therefore, *S*, *I*, and *Ru* are considered as latent states; and *A*, *R*, and *D* are considered as active states in the model. Each state is connected through transitional events. For example, the population in active cases state will transit to either recovery or fatality. Figure 1 shows all the states of the SEM model with the transitional events ($\mathcal {E}$) between the states. The feedback mechanism helps to simulate the spread based on latent states utilizing the active states.

The SIR model [34] aims to describe the behaviour of countries based on the states that vary as a function of time, *t*. With the known initial values for the states, this SIR model simulates the spread of the disease based on the parameters inferred from the epidemiological model. The differential equations for this model is defined as,

1 $$\begin{aligned} \frac{{\text {d}}S}{{\text {d}}t}= & {} \frac{-g(A,R,D)SI}{P}, \end{aligned}$$

2 $$\begin{aligned} \frac{{\text {d}}I}{{\text {d}}t}= & {} -(\gamma + \eta \beta )I+\frac{g(A,R,D)SI}{P}, \end{aligned}$$

3 $$\begin{aligned} \frac{{\text {d}}Ru}{{\text {d}}t}= & {} \eta \beta I_t, \end{aligned}$$

4 $$\begin{aligned} \frac{{\text {d}}A}{{\text {d}}t}= & {} \gamma I_t - (\beta + \gamma )A, \end{aligned}$$

5 $$\begin{aligned} \frac{{\text {d}}R}{{\text {d}}t}= & {} \beta A, \end{aligned}$$

6 $$\begin{aligned} \frac{{\text {d}}D}{{\text {d}}t}= & {} \delta A, \end{aligned}$$

where *g*(*A*, *R*, *D*) is a transmission rate function; $\beta$, $\gamma$, $\eta$, and $\delta$ are parameters.

The transmission rate function is defined to model the changes in behaviour of a population in response to reported and confirmed case numbers,

7 $$\begin{aligned} g(A,R,D) = \alpha _0 + \frac{\alpha }{[1+ (\omega _A A)^n]}, \end{aligned}$$

where $\alpha _0$ and $\alpha$ are residual and controllable transmission rate parameters, respectively. $\omega _A$ and *n* are the response weight and slope parameters, respectively (see Warne et al. [34] for details).

Realizations of the model are simulated using the Tau-leaping approximate stochastic simulation (TLASS) [15] method. Let $\varvec{\mathrm {X}}_t = [S_t, I_t, A_t, R_t, D_t, Ru_t]^\mathrm{T}$ be the state vector at time *t*. The next state vector $\varvec{\mathrm {X}}_{t+1}$ is simulated using TLASS based on the differential equations expressed in Eqs. (1,2,3,4,5,6)–(7). The algorithm starts the simulation with initial state vector defined as,

8 $$\begin{aligned} \varvec{\mathrm {X}}_0 = [P - \kappa A_0 \!-\! (A_0 \!+\! R_0 + D_0), \kappa A_0, A_0, R_0, D_0, 0]^\mathrm{T},\nonumber \\ \end{aligned}$$

where $\kappa$ is a parameter indicating the relative number of unobserved cases and *P* is the country’s population.

To generate simulations from the model defined by Eqs. (1,2,3,4,5,6)–(7) with initial conditions given in Eq. (8), a vector of nine unknown parameters, $\theta = [\alpha _0, \alpha , \beta , \gamma , \delta , n, \kappa , \eta ,\omega _A]$, needs to be inferred. Table 1 shows the list of all the parameters involved in the SIR model [34].

**Table 1:** List of parameters inferred in the SIR model using ABC-SMC [34]

Parameter	Description
$\alpha _0$	Residual transmission
$\alpha$	Regulatable transmission
$\beta$	Recovery rate
$\gamma$	Detection rate
$\delta$	Death rate
*n*	Response slope
$\kappa$	Initial relative latent infections
$\eta$	Latent recovery rate scale factor
$\omega _A$	Response weights

Inferring these parameters will allow us to interpret the detailed analysis of the complex interactions between the spread of COVID-19 and the community behaviour. Suppose $\mathcal {D}_i$ is observed data of active, recovered, and fatality cases from the JHU dataset for each country for a certain period. Using this observed data, posterior distributions for each country are sampled using an adaptive sequential Monte Carlo approach for approximate Bayesian computation (ABC-SMC) [13, 31, 33]. This approach propagates a set of samples, called particles, initially drawn from the prior distribution, through a family of intermediate distributions to ultimately obtain samples from the joint posterior distribution defined as,

9 $$\begin{aligned}&p(\theta \mid \mathcal {D}_i) \approx p(\theta \mid \rho (\mathcal {D}_i,\mathcal {D}_s) \le \epsilon ) \nonumber \\&\quad \propto \mathbb {P}(\rho (\mathcal {D}_i,\mathcal {D}_s) \le \epsilon ~\mid ~ \theta )~p(\theta ) \nonumber \\&\quad = p(\theta ) \int \mathbbm {1}_{(0, \epsilon ]} (\rho (\mathcal {D}_i,\mathcal {D}_s))s(\mathcal {D}_s \mid \theta )~\mathrm {d}\mathcal {D}_s, \end{aligned}$$

where $\theta$ is the unknown parameter, $\mathcal {D}_s \sim s(.\mid \theta )$ is the simulated data generated based on the initial parameter, $\rho (\mathcal {D}_i,\mathcal {D}_s)$ is a discrepancy metric, $\epsilon$ is the discrepancy threshold, and $p(\theta )$ is the prior distribution. $\mathbbm {1}_{(0, \epsilon ]}(\rho (\mathcal {D}_i,\mathcal {D}_s)) = 1$ if $\rho (\mathcal {D}_i,\mathcal {D}_s) \le \epsilon$ and $\mathbbm {1}_{(0, \epsilon ]}(\rho (\mathcal {D}_i,\mathcal {D}_s)) = 0$ otherwise.

ABC-SMC ultimately outputs *N* samples from the posterior distribution informed by the data for a given county. Each sample represents a plausible parameters combination given the reported case data for that country. The set of all samples for a country provides a discrete representation of the posterior distribution that can be used to quantify the uncertainty in these parameters given the data.

For the cluster analysis, we marginalize the $\eta$ parameter since Balasubramaniam et al. [5] demonstrate this nuisance parameter is unidentifiable. Therefore, we only consider the remaining eight parameters in the remainder of the paper. For more details on ABC-SMC and parameters, please refer to Warne et al. [34].

## 3-D clustering using nonnegative tensor factorization

The parameter inference of the SIR model using ABC-SMC as discussed in the previous section leads to the generation of eight parameters values for *M* countries. The parameters are inferred for each country for *I* particles (or samples) to represent uncertainties that arise due to dependencies on population. Since the parameters are inferred for different samples, they can quantify the uncertainty.

This representation establishes a multi-dimensional relationship among country, parameter, and particle. Usage of a traditional matrix model is not suitable to represent this type of three-dimensional relationship. Any such attempt will lead to information loss [10]. For example, the simplest approach is to average a parameter value of each country over all the particles. Though it seems valid, the information loss happening here impacts the results in terms of cluster quality and their interpretation. On the other hand, a tensor model [22], which is an extension of the matrix for high dimensions, can represent multi-dimensional relationships among country, parameter, and particles effectively.

### Problem formulation

Let $\mathcal {C} =\{c_1,c_2,\ldots ,c_M\}$ be a set of *M* countries and $\mathcal {P} = \{p_1,p_2,\ldots ,p_N\}$ be a set of *N* parameters representing each countries’ responses to COVID-19 modelled with the SIR.^2^$\mathcal {D} =\{d_1,d_2,\ldots ,d_I\}$ be a set of *I* particles, such that each country and parameter is associated with *I* particles.

The traditional clustering methods such as KMeans [20], DBScan [14], and NMF [27] accept the input data as a matrix representation. While the matrix representation is capable of representing the relationship between country and parameter, it is incapable of representing the relationship between country, parameter, and particle. This will lose some information and hence the quality of clustering solution will be affected.

**Fig. 2:**
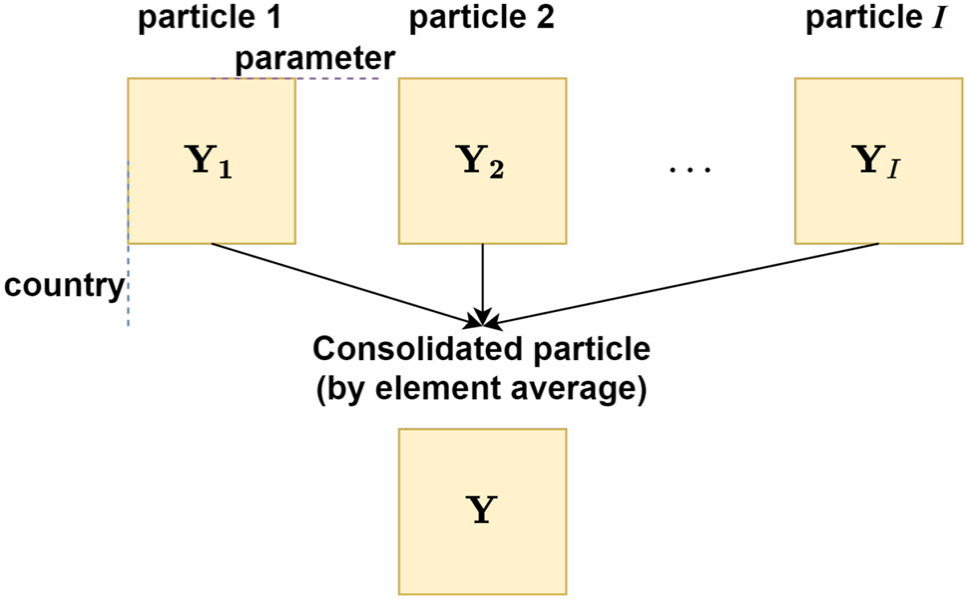
Example of traditional consolidated matrix representation. Here multiple matrices representing country $\times$ parameter (i.e. $\mathcal {C} \times \mathcal {P}$) over multiple particles are consolidated by element average to derive a single matrix $\varvec{\mathrm {Y}}$

Let us consider an example. Suppose the country parameter relationship (i.e. $\mathcal {C} \times \mathcal {P}$) at *i*th particle is represented as a matrix, $\varvec{\mathrm {Y}}_i \in \mathbb {R}^{M \times N}$. It requires *I* number of matrices to represent country parameter relationship at different particles as shown in Fig. 2. Since a single clustering solution from multiple matrices is not possible, consolidation of matrices is the straightforward approach.

From *I* matrices, a consolidated matrix can be calculated as an element-wise average as,

10 $$\begin{aligned} \varvec{\mathrm {Y}} = \frac{\varvec{\mathrm {Y}}_1 + \varvec{\mathrm {Y}}_2 + \cdots + \varvec{\mathrm {Y}}_i}{I}. \end{aligned}$$

Alternatively, the consolidation can be calculated as a median. However, this approach may fail to capture the variations and uncertainty inherent in the data correctly. Another simplified approach is the concatenation of country, parameter, and particle into a single vector. This will represent each country as a vector of length $N \times I$. Suppose there are 8 parameters and 500 particles, this will create a vector of length 4000 to represent each country. The objective of clustering in this paper is to cluster the countries at different periods to see how these countries evolved during this period. The concatenation is meaningful for this case as it can cluster the countries. Assume we treat each sample (particle) from the posterior as a vector and apply a clustering algorithm for our objective. This approach will generate clusters containing the same country from different samples (particle) appearing in different clusters. Such a result is not ideal for the problem defined in this research. In the previous work of Warne et al. [34], the ABC posterior particle with the lowest discrepancy with the data is used as a point estimate to reduce the dimensions. The discrepancy of a particle represents a measure of distance between the data and a stochastic simulation of our model using that particular particle. Looking at the distribution of the discrepancy measure can be informative for ABC inference, for example, a distribution with many larger discrepancy particles will relate to posterior predictive distributions with much wider uncertainty regions with the actual data potentially in the tails. While clustering can be done on this concatenated and point estimated data, they suffer from the curse-of-dimensionality or lose the explainability, as elaborated later in the results section.

Hence, a better way of representing this three-dimensional data is inevitable. In this paper, we adapt the tensor model as an effective solution.

### Tensor model representation

**Fig. 3:**
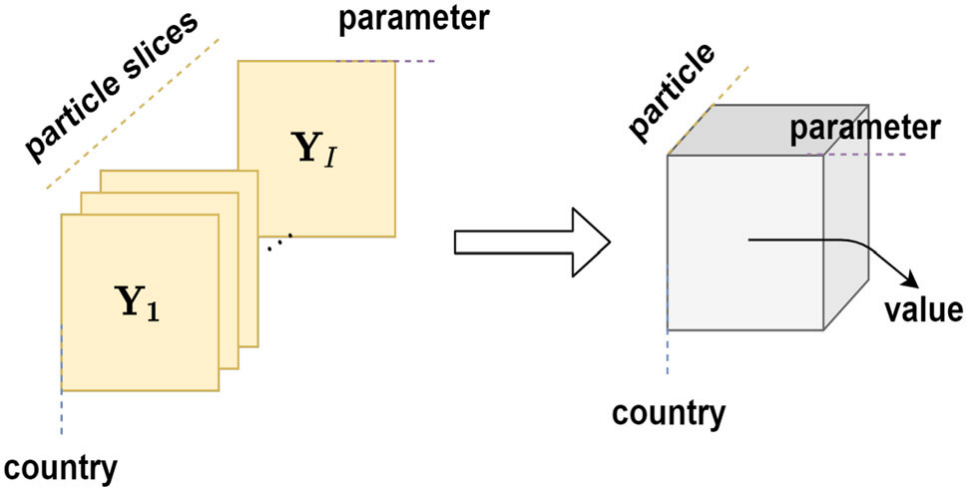
Example of tensor representation. Instead of consolidation, multiple matrices encoding country $\times$ parameter over multiple particles are represented as a three-dimensional tensor, where particle is the third dimension

The multi-dimensional relationships that exist between the country, parameter, and particle are captured through a tensor representation as shown in Fig. 3. In tensor representation, instead of consolidation, the matrices are stacked. This stacking of multiple matrices is called slices of the tensor [22].

Consider country $c_1$ has a value = 1 for parameter $p_3$ at particle $d_3$. For this relationship, an entry in tensor will be populated as $\varvec{\mathcal {X}}_{1,3,3} = 1$, where $\varvec{\mathcal {X}}$ is a tensor of size $\mathbb {R}^{M \times N \times I}$ and each dimension (country, parameter, or particle) is called mode. Similarly, all the entries can be populated by preserving the relationships among the modes. Now the objective is to identify groups (i.e. clusters) of countries that show a similar response to COVID-19.

### Nonnegative tensor factorization (NTF)

NTF [9] is a dimensionality reduction technique that learns the latent dependencies among multiple modes and decomposes a large tensor into multiple smaller factor matrices with each matrix representing a mode in reduced dimensions. Clustering can be performed on a factor matrix to find sub-groupings for a mode. The dominating value of each row in a factor matrix represents cluster membership of the mode [26].

One advantage of using NTF in this research is its ability to learn the country clusters as well as to learn the parameter and particle patterns. Parameter and particle patterns will help to interpret the characteristics of a country cluster. Each factor matrix will learn this latent relationship because of the NTF’s capability to capture associations between values of the dimensions (i.e. country, parameter, and particle) through factorization. This kind of interpretation is useful, especially in a pandemic situation such as COVID-19, to understand the reasons for countries being clustered together.

A 3-D tensor $\varvec{\mathcal {X}}$ can be factorized into three factor matrices $\varvec{\mathrm {C}}$, $\varvec{\mathrm {P}}$ and $\varvec{\mathrm {D}}$ representing country, parameter, and particle modes, respectively. The column-wise outer product of factor matrices is approximately equal to the original tensor defined as follows,

11 $$\begin{aligned} \varvec{\mathcal {X}} \cong [\![\varvec{\mathrm {C}}, \varvec{\mathrm {P}}, \varvec{\mathrm {D}} ]\!]= \sum _{r=1}^{R} \varvec{\mathrm {c}}_{r} \circ \varvec{\mathrm {p}}_{r} \circ \varvec{\mathrm {d}}_{r}, \end{aligned}$$

where $\varvec{\mathrm {C}} \in \mathbb {R}^{(M \times R)}$, $\varvec{\mathrm {P}} \in \mathbb {R}^{(N \times R)}$ and $\varvec{\mathrm {D}} \in \mathbb {R}^{(I \times R)}$ are factor matrices with $R$ hidden features (also called as Rank), $\circ$ indicates outer product, $R \in \mathbb {Z}_{+}$. $\varvec{\mathrm {c}}_{r}$, $\varvec{\mathrm {p}}_{r}$ and $\varvec{\mathrm {d}}_{r}$ are the $r^{th}$ column of $\varvec{\mathrm {C}}$, $\varvec{\mathrm {P}}$ and $\varvec{\mathrm {D}}$, respectively. This outer product of columns of factor matrices represents rank-1 tensor product that preserves the association among the dimensions.

Equation (11) can also be represented as the element-wise inner product of factors to approximate the elements of the original tensor as follows,

12 $$\begin{aligned} \varvec{\mathcal {X}}_{m,n,i} \cong \varvec{\mathcal {\hat{X}}}_{m,n,i} = \sum _{r=1}^{R}c_{mr}~.~p_{nr}~.~d_{ir} \end{aligned}$$

where $\varvec{\mathcal {X}}_{m,n,i}$ is $(m,n,i)^{th}$ element in $\varvec{\mathcal {X}}$; $\varvec{\mathcal {\hat{X}}}_{m,n,i}$ is approximated value of $\varvec{\mathcal {X}}_{m,n,i}$; $c_{mr}$, $p_{nr}$, and $d_{ir}$ are elements of $\varvec{\mathrm {C}}$, $\varvec{\mathrm {P}}$ and $\varvec{\mathrm {D}}$, respectively.

NTF is a non-convex optimization problem that aims to learn the values for each factor matrix. The factor matrices are learned through optimization minimization and the objective function is defined as a minimization of Euclidean distance between the original tensor and the approximated tensor constructed using factor matrices.

The optimization minimization problem of the well-known CANDECOMP/PARAFAC (CP) factorization [9] can be defined as,

13 $$\begin{aligned} \min _{\varvec{\mathrm {C \ge 0,P \ge 0, D \ge 0}}}f(\varvec{\mathrm {C}},\varvec{\mathrm {P}},\varvec{\mathrm {D}}) = \left\Vert \varvec{\mathcal {X}} - [\![\varvec{\mathrm {C}}, \varvec{\mathrm {P}}, \varvec{\mathrm {D}} ]\!]\right\Vert ^2. \end{aligned}$$

Equation (13) is equivalent to the following element-wise optimization problem,

14 $$\begin{aligned} {\begin{matrix} &{}\min _{\varvec{\mathrm {C \ge 0,P \ge 0, D \ge 0}}}f(\varvec{\mathrm {C}},\varvec{\mathrm {P}},\varvec{\mathrm {D}}) \\ &{}\quad =\sum _{m=1}^{M}\sum _{n=1}^{N}\sum _{i=1}^{I} \left\Vert \varvec{\mathcal {X}}_{m,n,i} - \varvec{\mathcal {\hat{X}}}_{m,n,i}\right\Vert ^2. \end{matrix}} \end{aligned}$$

**Fig. 4:**
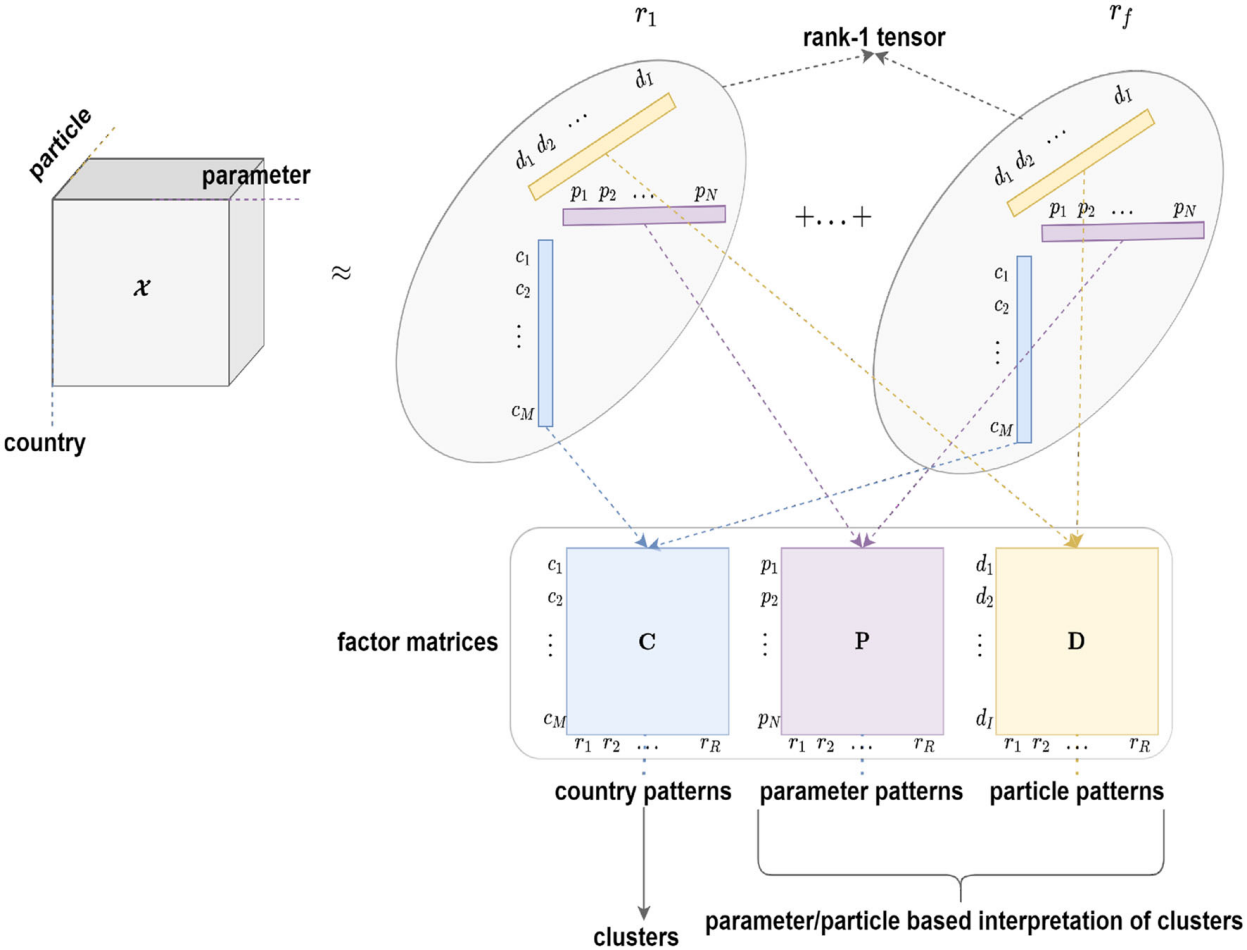
Overall process of NTF for clustering and interpretation. A 3-D tensor is factorized into 3 factor matrices $\varvec{\mathrm {C}},\varvec{\mathrm {P}}$, and $\varvec{\mathrm {D}}$ representing the latent features of countries, parameters, and particles, respectively, after learning dependencies among the modes. Each factor matrix can become input to clustering the countries and interpreting the parameter and particle patterns

To simplify the optimization process, the tensor model will be matricized (i.e. a process of representing a tensor in a matrix form). For the matricized tensor, the optimization minimization problem is defined as,

15 $$\begin{aligned} \min _{\varvec{\mathrm {C \ge 0,P \ge 0, D \ge 0}}}f(\varvec{\mathrm {C}},\varvec{\mathrm {P}},\varvec{\mathrm {D}}) = \frac{1}{2}\left\Vert \varvec{\mathrm {X_1}} - \varvec{\mathrm {C}}\Big (\varvec{\mathrm {P}} \odot \varvec{\mathrm {D}}\Big )^\mathrm{T}\right\Vert ^2, \end{aligned}$$

where $\varvec{\mathrm {X_1}}$ is the mode-1 matricization of $\varvec{\mathcal {X}}$, $\odot$ is Khatri-Rao product. Mode-1 matricization means unfolding the tensor into a matrix by fixing mode-2 and mode-3. For example, a tensor of size $\mathbb {R}^{(4 \times 5 \times 6)}$ will be unfolded into a matrix of size $\mathbb {R}^{(4 \times 30)}$. The mode-1 matricization of a third-order tensor will adjacently place six ($4 \times 5$) matrices.

The factor matrices are learned using a common factorization algorithm such as Alternating Least Square (ALS) [28], Gradient Descent [35], or Coordinate Descent (CD) [19]. The CD-based factorization algorithms have proven to be scalable as well as accurate. In this paper, a recent CD-based algorithm, Saturating Coordinate Descent [5], is used to solve Equation (15). Please refer to [5] for derivations and update rules.

It can be observed from Eq. (15) that the factor matrices are learned and their inner product is used in the generation of an approximate solution. Because of the inner product between the modes, the value of a factor matrix learned during the factorization will be a representation of the association between the modes. Moreover, the inner product between the columns indicates the association between the columns of factor matrices. Note that each column of a factor matrix can be considered a hidden feature. The values of a feature will specify the weightage of the feature in learning the element values in the original tensor.

Equation (11) represents the summation of rank-1 tensors. Let us consider an example of a rank-1 tensor as shown in Fig. 4. Here, three columns indicate features from three modes. The inner product of three features (i.e. vectors) will help to generate a single element (i.e. a value in the tensor). This means the association of three modes captured in factor matrices will be the approximate representation of the original data point. This also shows that each rank-1 tensor is one pattern of modes that captures their association. Therefore, this approach can provide a detailed interpretation.

### Interpretation

**Fig. 5:**
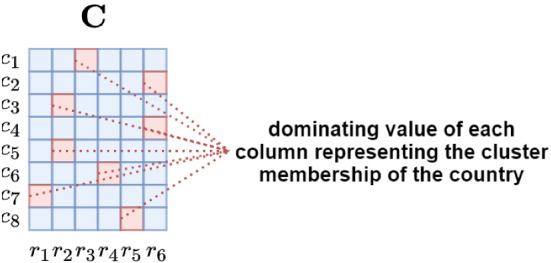
Determining the six clusters containing similar countries based on the factor matrix $\varvec{\mathrm {C}}$

The NTF process yields three factor matrices as output. A factor matrix represents latent features of a mode. The number of latent features is decided by the chosen rank value (the number of latent features $==$ *R*). A factor matrix can be used to generate clusters in two ways. (1) If a large value is chosen for the rank value, then each latent feature is treated as a variable. The factor matrix becomes input to a clustering algorithm such as KMeans and the required clusters are identified. (2) If a rank value is chosen as the required cluster number, each latent feature represents a cluster. As shown in Fig. 5, the dominant value in a row decides the cluster membership in this scenario.

We use the second approach as our objective is to aid to interpretability via clustering. Whereas, the first approach, i.e. clustering the latent features, attracts complexities such as distance concentration problem and lack of easy visualization for interpretation. This is extensively discussed in the Results section.

As shown in Fig. 5, let us consider that there are 8 countries (*M* = 8) and the rank (i.e. number of clusters) is set to 6 (*R* = 6) for factor matrix $\varvec{\mathrm {C}}$. While each column indicates the most representative countries in a cluster, each row indicates the low-rank representation of a country. While each country appears in all clusters, it becomes the member of the cluster with the highest value. In other words, the dominating value of each row represents the cluster membership of the country. Countries that have the same dominating cluster indicate that they are similar and hence will be considered as members of the same cluster.

While we use the factor matrix $\varvec{\mathrm {C}}$ to identify the country clusters, the other factor matrices are used to characterize the country clusters. Factor matrices $\varvec{\mathrm {P}}$ and $\varvec{\mathrm {D}}$ represent the parameters and particles, respectively, with 6 latent features. Because the factor matrices are learned through associations as shown in Fig. 4, the column 1 of each of the $\varvec{\mathrm {P}}$ and $\varvec{\mathrm {D}}$ matrices is associated with the cluster 1 (i.e. column 1) of $\varvec{\mathrm {C}}$. For a detailed understanding of countries grouped in the same cluster, the features of parameters and particles are used to characterize the cluster. Therefore, for these two factor matrices $\varvec{\mathrm {P}}$ and $\varvec{\mathrm {D}}$, the values of each column are ranked instead of identifying the dominating value of each row as in $\varvec{\mathrm {C}}$. This is done so that the importance of these features can be characterized for each country cluster.

**Fig. 6:**
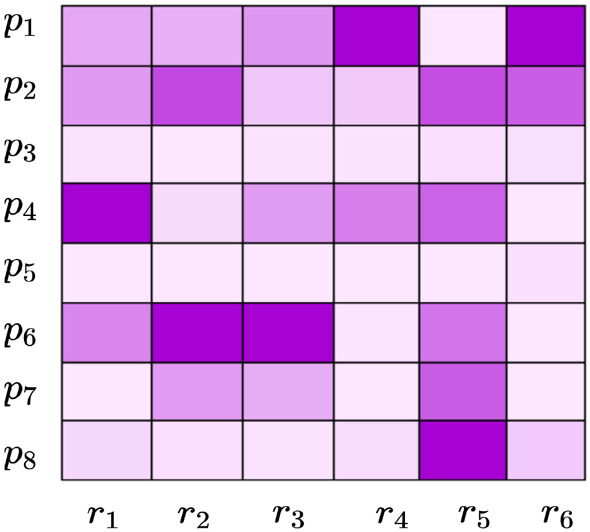
Interpretation of country clusters using the factor matrix $\varvec{\mathrm {P}}$ which represents the parameter patterns. The darker the colour in each column, the higher the value of the feature, indicating the characteristics of that column

Let us consider the factor matrix $\varvec{\mathrm {P}}$ with 8 parameters represented by 6 features, as shown in Fig. 6. Here, the values in each column will define the characteristics of countries clusters identified using $\varvec{\mathrm {C}}$. Relating $r_2$ in $\varvec{\mathrm {C}}$ (Fig. 5) and $\varvec{\mathrm {P}}$ (Fig. 6), the characteristics of cluster 2 ($r_2$ in $\varvec{\mathrm {C}}$) with countries $c_3$ and $c_5$ can be identified as a cluster with higher $p_6$ and moderate $p_2$ ($r_2$ in $\varvec{\mathrm {P}}$). Similarly, the particle features can also be used to interpret the characteristics of country clusters. The importance of interpreting country clusters using relationships with the particles is to have more explainability on the parameters. With NTF, we can compare cluster characteristics with respect to parameters and find which particles are contributing to such characteristics of parameters. This is especially important to understand how the lower and higher discrepancy particles impact parameter inference.

## Results

We conducted experiments to cluster the countries according to their similar early responses to the COVID-19 outbreak. A 3-D clustering was applied to the Bayesian analysis outcome of a SIR model represented as a tensor model. We attempt to answer the following questions related to the spread of COVID-19.

- **Q1**. What are the characteristics of the country clusters?
- **Q2**. Are there any countries that exhibit similar patterns in the early breakout period?
- **Q3**. Which countries in April 2020 are similar to China? What is the trajectory of China and is there any other country that followed their suit?

### Dataset

We use the same datasets from JHU as used in Warne et al. [34]. The JHU observatory observes each country’s active, recovered, and fatality cases as a time series. To understand the global response behaviour trends, three datasets from three different periods (Jan–March, Jan–April, and Jan–June of 2020) are used. These periods are selected to represent the exponential growth, peak, and recovery stages of the first wave of the COVID-19 pandemic for many countries. The Bayesian analysis is run on these three datasets to infer 8 parameters with 500 particles using ABC-SMC [34], and the output is modelled as a tensor (Table 2).

**Table 2:** Dataset statistics

Dataset	Time period	# countries	Tensor size
March (T1)	22 Jan to 30 Mar 2020	98	$98 \times 8 \times 500$
April (T2)	22 Jan to 06 Apr 2020	121	$121 \times 8 \times 500$
June (T3)	22 Jan to 09 June 2020	158	$158 \times 8 \times 500$

### Evaluation measures and identification of number of clusters

To demonstrate the advantages of using tensor model representation and NTF, two evaluation measures, Silhouette Score (SS) [30] and Pattern Distinctiveness (PD) [5] are used. Silhouette Score measures the inter and intra-cluster similarity. A high Silhouette score is desirable as it presents a high intra-cluster similarity (i.e. members of a cluster are close to each other) and low inter-cluster similarity (i.e. members of different clusters are distant to one another). The SS value ranges from $-\,1$ to 1, 1 being the best score. PD calculates the cosine similarity of each cluster with other clusters. This measures how distinctive the patterns or clusters are. Larger PD means the patterns identified are more unique and hence a larger value is desirable. The PD value ranges from 0 to 1, 1 being the best score. PD is calculated as follows,

16 $$\begin{aligned} PD = cosine(\varvec{\mathrm {c}}_m,\varvec{\mathrm {c}}_r), \forall ~ m,r \in [1,R], m\ne r, \end{aligned}$$

where ${\text {cosine}}(\varvec{\mathrm {c}}_m$, $\varvec{\mathrm {c}}_r)$ indicates the cosine similarity of *m*th and *r*th column of a factor matrix $\varvec{\mathrm {C}}$.

The information loss in clustering methods such as KMeans [20], DBScan [14], and NMF [27] can be quantified using measures such as H-score [36] and AAHR [6] using the ground-truth class labels for clusters [32]. Since we do not have ground-truth information on these datasets, the information loss can only be quantified using intrinsic measures such as SS and PD.

**Table 3:** Clustering performance

Metric	Technique	T1	T2	T3
SS	KMeans—AVG	0.3379	0.1612	0.3236
	DBScan—AVG	$-\,0.2036$	$-\,0.4865$	$-\,0.5356$
	NMF—AVG	0.2821	0.2028	0.3077
	KMeans—PE	0.2543	0.2750	0.3196
	DBScan—PE	$-\,0.2194$	$-\,0.1622$	$-\,0.3816$
	NMF—PE	0.3360	0.2733	0.2981
	KMeans—CON	0.0044	0.0110	0.0324
	DBScan—CON	–	–	–
	NMF—CON	0.3415	0.2939	0.3150
	NTF	**0.3483**	**0.2947**	**0.3308**
PD	KMeans—AVG	–	–	–
	DBScan—AVG	–	–	–
	NMF—AVG	**0.4346**	0.4980	0.4329
	KMeans—PE	–	–	–
	DBScan—PE	–	–	–
	NMF—PE	0.4104	0.4300	0.3735
	KMeans—CON	–	–	–
	DBScan—CON	–	–	–
	NMF—CON	0.4133	0.4959	0.4303
	NTF	0.4319	**0.5451**	**0.4482**

Bold value indicates the best clustering performance. The higher the value better the result

The main challenge with a clustering approach is the identification of an optimal number of clusters. Therefore, sensitivity analysis is conducted to identify the optimal number of clusters. The NTF is run for the ranks ranging between 2 and 8, and the rank, at which the SS and PD are highest, is selected. If SS and PD are maximum at different rank values, a sensitivity analysis based on approximation error [5] can be used as a guide to decide the optimal number of ranks. A lower approximation error indicates better quality factorization. The reason for setting the maximum rank to 8 is because the number of parameters is 8. Setting a rank more than any of the mode sizes will lead to learning repeated patterns [38].

### Clustering results

Table 3 presents the results of the evaluation measures for NTF and other traditional clustering methods such as KMeans [20], DBScan [14], and NMF [27]. For the datasets representing three different periods (T1, T2, and T3), three NTF solutions are generated. Informed by the sensitivity analysis, the optimal rank/number of clusters is found as 6, 6, and 7 for the period March, April, and June, respectively. To demonstrate the importance of including particles as the third order in NTF, three variations of matrix representation (i.e. AVG—average, PE—point estimate, and CON—concatenation) as discussed in Sect. 4.1 are generated and used by KMeans, DBScan, and NMF clustering methods.

While the high-dimensional data representation by concatenation can include particle information, it suffers from the curse-of-dimensionality leading to the distance concentration problem (i.e. difficulty in measuring the nearest and farthest points using distance measures) [37]. Hence, KMeans—CON, and DBScan—CON methods show the worst performance (i.e. near zero and negative SS scores) as shown in Table 3. DBScan—CON did not work for this high-dimensional data and fails to identify any meaningful clusters (as indicated by negative SS scores) and assigns all the data points to a singleton cluster. Comparatively, NMF shows a better clustering performance on the high-dimensional data because it first projects the data as low-dimensional features and finds the cluster membership in reduced-space representation. However, the NMF—CON output with a 4000 (8 parameters $\times$ 500 particles) mode size is not easy to visualize or interpret.

Overall, the PE results are superior to the simple AVG matrix representation as shown in Table 3. However, this does not outperform the NTF clustering. These results demonstrate the utility of NTF for a high-dimensional cluster analysis for the interpretation of COVID-19 response parameters. It is evident from Table 3 that the inclusion of particles as the third dimension has resulted in NTF outperforming other clustering methods.

Since NMF and NTF are the only methods capable of representing high-dimensional data to a low-rank representation, the patterns in the projected lower-rank data can be derived. Hence PD is applicable only for these two methods. Results show that NTF is 2–7% better than NMF. Moreover, given the small size of the data ($158 \times 8 \times 500$), NTF is not computationally demanding and the run-time of NTF is not significantly higher than NMF.

### Cluster interpretation

**Fig. 7:**
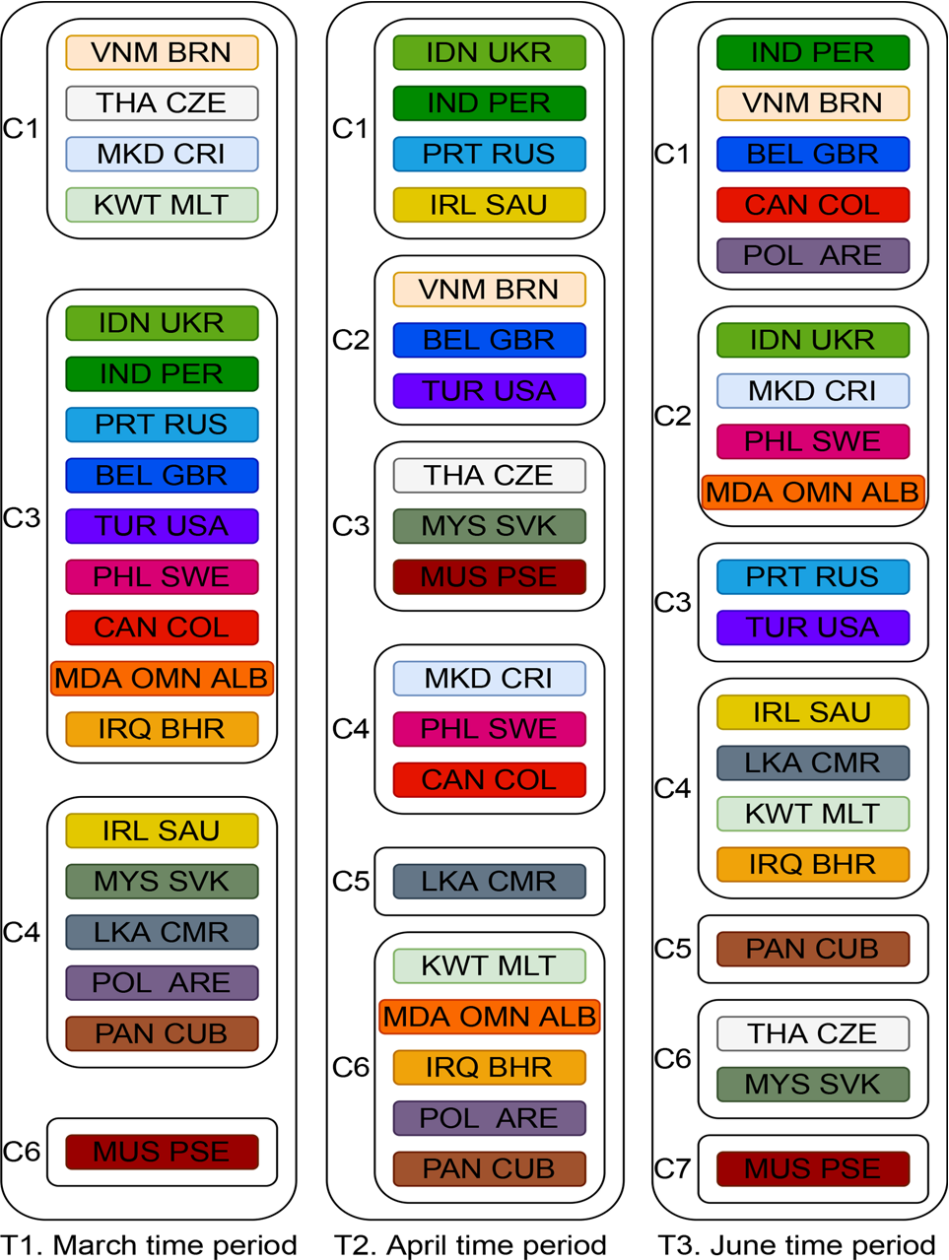
Countries grouped together in all three datasets. Each colour indicates the set of countries that travel together from T1 to T2 to T3. Some clusters are not included in the figure as they do not travel together with any other countries for all three datasets

Tables 4, 5, and 6 show the countries^3^ clustered together in different periods using NTF. Figure 7 shows the movement pattern of all the countries grouped in all three periods. Each colour indicates a set of countries within each cluster that exhibit similar characteristics in the form of 8 parameters. For instance, a set of countries from C6 in T1 moves to C3 in T2 and to C2 in T3. This indicates that these countries follow similar approaches of responding to the spread of COVID-19.

**Fig. 8:**
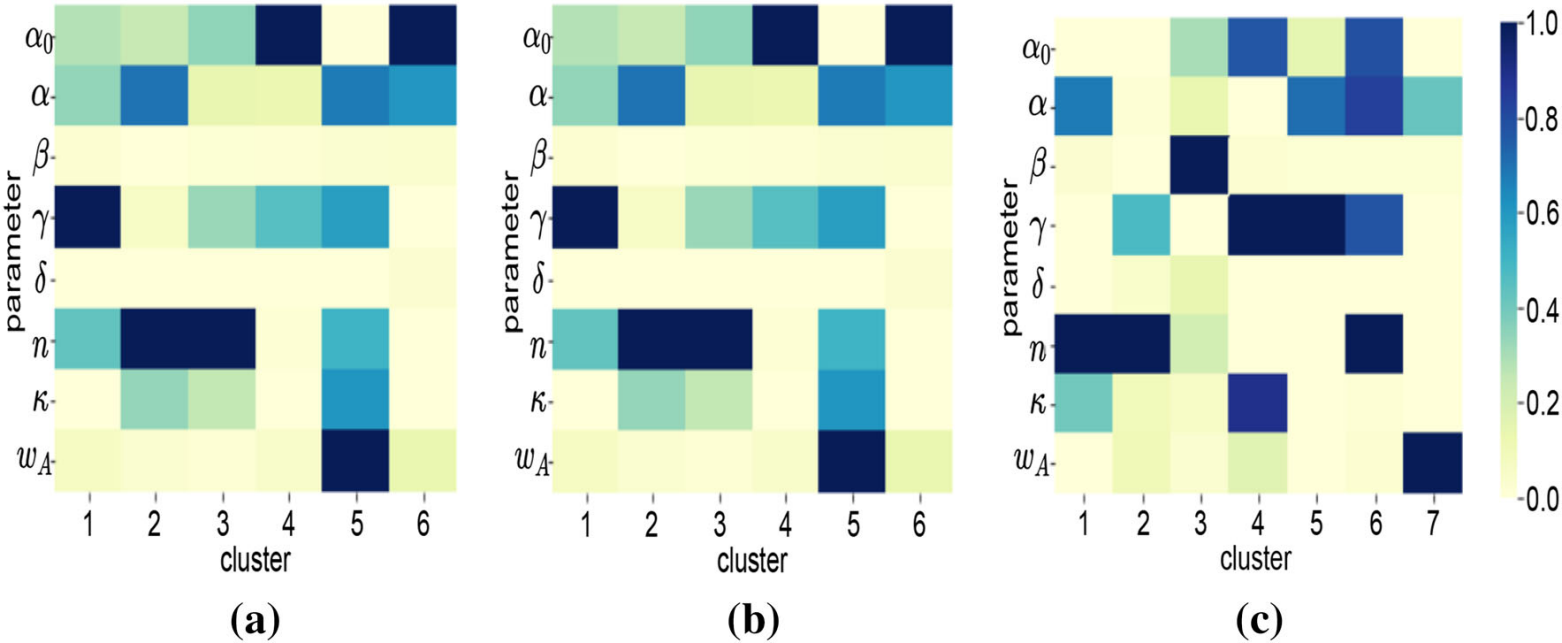
Characteristics of clusters w.r.t. parameters using NTF: **a** T1(March time-period); **b** T2 (April time-period); and **c** T3 (June time-period)

To interpret the characteristics of countries in each cluster, parameter and particle factor matrices are used. Figure 8 shows the characteristics of each cluster for different periods. As discussed in Sect. 4.4, each column in Fig. 8 represents the characteristics of clusters in terms of parameters. For instance, cluster 1 in Fig. 8a has high $\gamma$. This indicates that countries clustered in Cluster 1 during the January–March period had a high detection rate.

**Table 4:** Country clusters for T1 (March period)

Cluster	Countries
1	AND ARG BFA BGR BRA BRN CRI CZE DNK ECU GRC JOR KAZ KWT LUX MKD MLT QAT THA TUN TWN URY UZB VNM ZAF
2	AUT CHE CHL ESP FIN ISL LBN LTU LVA MAR PAK SVN
3	ALB AUS BEL BHR CAN COL CYP DZA GBR HRV IDN IND IRQ ISR ITA JPN MDA OMN PER PHL PRT RUS SGP SWE TUR UKR USA
4	ARE ARM AZE BIH CMR CUB DEU DOM EST GHA HUN IRL KHM LKA MYS NLD NOR PAN POL SAU SRB SVK
5	CHN EGY IRN KOR MEX SEN
6	AFG CIV FRA HND MUS NGA NZL PSE ROU VEN

**Table 5:** Country clusters for T2 (April period)

Cluster	Countries
1	BGD CHL DJI ECU GHA GIN IDN IND IRL KGZ MDG MLI NLD PER PRT RUS SAU SLV SRB UKR UZB
2	BEL BRN DOM FRA GBR HRV IRN ITA JOR KEN KHM MNE SVN TUR TWN URY USA VEN VNM
3	AZE BFA BIH CHN COD CZE DZA EST FIN GTM HUN ISL ISR LBN MUS MYS NER NGA NOR NZL PRY PSE RWA SEN SVK THA TTO TUN ZAF
4	ARG BRA CAN COL CRI GRC LTU MEX MKD PAK PHL ROU SWE
5	AND ARM AUS AUT CHE CMR DEU ESP JPN KAZ KOR LKA MAR
6	AFG ALB ARE BGR BHR BOL CIV CUB CYP DNK EGY HND IRQ KWT LUX LVA MDA MLT OMN PAN POL QAT SGP

**Table 6:** Country clusters for T3 (June period)

Cluster	Countries
1	AFG ALB ARM BFA BIH BOL COD COG CPV CRI DJI DNK EGY GNQ GUY IDN KAZ KGZ LBR MDA MKD MMR MOZ MRT NGA OMN PHL PRY ROU SLE SLV SOM STP SWE SWZ TJK UKR UZB
2	BGR CYP EST HND ITA JAM KEN LBN MLI MNG MUS PSE RWA SUR SYR TGO TUN TZA UGA VEN
3	AZE BHR CHL CMR DOM DZA ETH IRL IRQ ISL JPN KWT LKA MAR MDG MEX MLT NZL SAU SEN URY ZAF
4	ARE BEL BEN BGD BHS BRA BRN CAN CHE COL COM FRA GBR IND IRN KHM MNE NLD PAK PER POL SGP TTO VNM
5	AND AUS AUT CHN CZE GIN GNB GRC HRV KOR LTU LUX LVA MWI MYS NER NIC SRB SVK SVN TCD THA TWN ZMB
6	CAF CIV CUB FIN GAB GHA GTM HUN MDV PAN SDN SSD YEM
7	ARG DEU ECU ESP HTI ISR JOR LBY NOR NPL PRT QAT RUS TUR USA

**Fig. 9:**
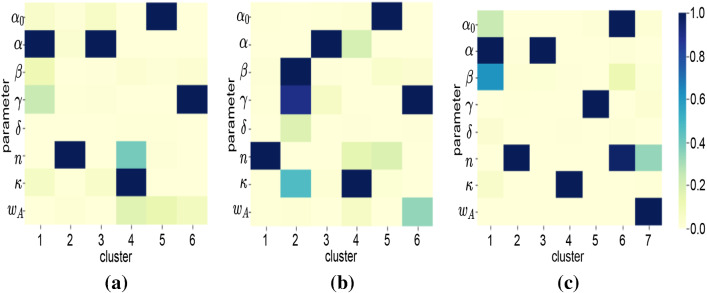
Characteristics of clusters w.r.t. parameters using NMF: **a** T1(March time-period); **b** T2 (April time-period); and **c** T3 (June time-period)

**Fig. 10:**
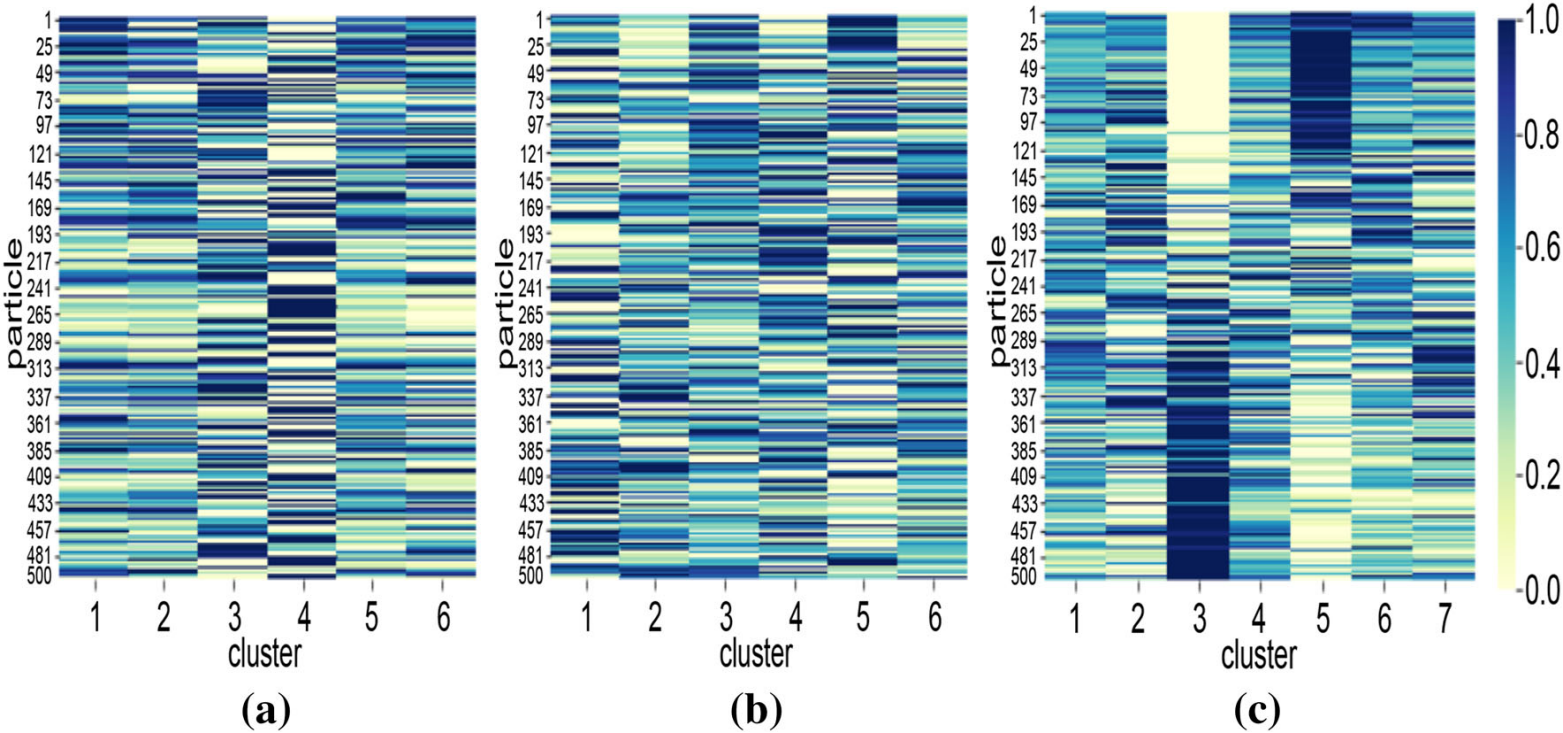
Characteristics of clusters w.r.t. particles: **a** T1 (March period); **b** T2 (April period); and **c** T3 (June period)

This answers the question Q1, and similarly, many interpretations can be made about clusters using their characteristics with respect to parameters. Some of the key insights about the trajectories of particular countries that can be identified using the cluster characteristics include:

- Three countries (MDA OMA ALB) are the only countries grouped in the same clusters in all three periods. They are grouped in Clusters 3, 6, and 1 in T1, T2, and T3, respectively.
- Many countries are grouped together in Cluster 3 of T1 (Fig. 4). But these countries split into T2 and T3. For example, [IDN, UKR, IND, PER, PRT, and RUS] are moving from Cluster 3 in T1 to Cluster 1 in T2. But [TUR, USA, BEL, and GBR] are moving from Cluster 3 in T1 to Cluster 2 in T2. Interestingly, [TUR, USA, PRT, and RUS] joins again in Cluster 7 in T3. This indicates that a different response strategy is practised by these countries while transiting from T1 to T2 to T3.
- Using Fig. 8, we can infer the characteristics of these four clusters.
  - Cluster 3 of T1: sudden change in behaviour before and after implementing response strategies (high *n*), delayed response (low $\omega _A$), medium detection rate (medium $\gamma$), medium initial spread (medium $\kappa$).
  - Cluster 1 of T2: more gradual change in behaviour before and after implementing response strategies (medium *n*), delayed response (low $\omega _A$), low detection (low $\gamma$), low initial spread (medium $\kappa$).
  - Cluster 2 of T2: sudden change in behaviour before and after implementing response strategies (high *n*), delayed response (low $\omega _A$), high detection (high $\gamma$), high initial spread (high $\kappa$).
  - Cluster 7 of T3: slow change in behaviour before and after implementing response strategies (low *n*), timely response (high $\omega _A$), low detection (low $\gamma$), low initial spread (low $\kappa$), high residual transmission rate (high $\alpha _0$).
  Fig. 7 shows all such movements which answer the question Q2.
- While the particle patterns as shown in Fig. 10 looks random, some clusters show distinct patterns. For instance, cluster 3 in T3 (Fig. 10c) is characterized by parameters inferred from particles with higher discrepancy with the data, whereas cluster 5 in T3 is characterized by parameters inferred from particles with lower discrepancy with the data. If particles are not included in the analysis, and if a point estimate based on low discrepancy is used, we will not be able to find the clusters that are formed based on parameters inferred by particles with higher discrepancy. This can be learned by interpreting the parameter patterns from NMF—PE as shown in Fig. 9c. NMF—PE is not able to identify any cluster with similar characteristics as cluster 3 (i.e. the parameters inferred from particles with high discrepancy) of T3 which is identified using NTF (Fig. 8c). Therefore, understanding the clustering and knowing how the countries are grouped along with the discrepancy information adds more value to the interpretation. Moreover, the clusters in Fig. 8 can quantify/characterize each cluster with multiple parameters but the clusters in Fig. 9 quantify/characterize each cluster with very few parameters. This minimizes the ability to interpret the clusters of countries. For example, cluster 1 in Fig. 8a is characterized by a higher detection rate with a moderate response, regulatable transmission, and residual transmission rates. On the other hand, cluster 6 in Fig. 9a is only characterized by a higher detection rate.

To interpret KMeans and DBScan clustering, we must visualize the distribution of each parameter with respect to each cluster. Therefore, for 8 parameters and 6 clusters we need to plot 48 distribution plots to understand the clusters. While it is too much to show in the paper, it is also not easy to interpret them. However, the factorization-based techniques NMF and NTF generating lower-dimensional features allow us to easily understand the cluster with just 48 scalar points as shown in Figs. 8 and 9.

**Fig. 11:**
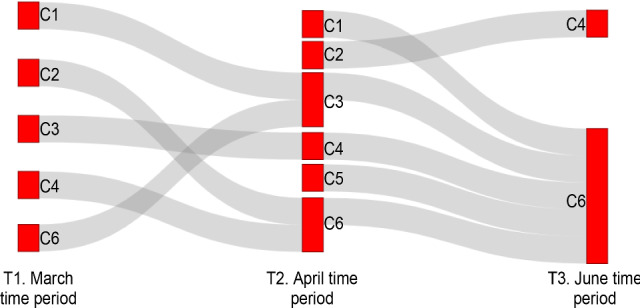
Cluster to cluster similarity based on characteristics. Similarity score of 0.5 is set as threshold. For example, the connection between C1 of T1 to C3 of T2 means that C1 of T1 is similar to C3 of T2

While Fig. 7 connects countries that move together in clusters through different periods, it does not connect clusters based on similarity. Connecting clusters based on similarity will help to understand how countries shift from one cluster to another based on characteristic similarity. This will enable us to get some insights into countries’ behaviour. Since clustering is independently carried out for each dataset, there is no one-to-one association between the clusters of different datasets. Therefore, we first try to find a one-to-one match for each cluster from different datasets using cosine similarity. Fig. 11 shows the connections between clusters that have at least 50% similarity. For example, clusters C1 and C6 of T1 are at least 50% similar to C3 of T2.

Let us consider an example of the country China. China is a country that has imposed strict restrictions to contain the virus and it has shown to be effective to date without a second wave [1]. In T1 China (CHN) is in cluster 5 which is characterized by very high $w_A$ with moderate $\alpha$, $\gamma$, $\kappa$, and *n* (refer to Fig. 8a). This indicates the response is very quick and the transmission is regulatable. Surprisingly, in T2 there exist no clusters with these characteristics. This gives us an important message that if countries have followed the characteristics of CHN in T1, the second wave might have been avoided. This provides insights into Q3. Similarly, many such insights can be elicited.

**Fig. 12:**
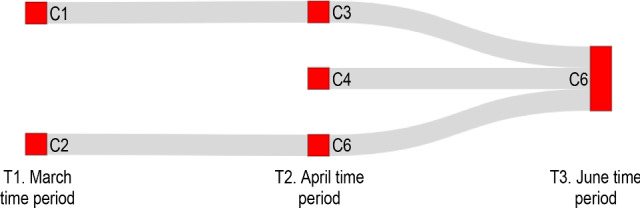
Cluster to cluster similarity based on characteristics. Similarity score of 0.8 is set as threshold

If we set a high cosine similarity threshold say 80%, only a few characteristics are repeated among the clusters from the different periods. For example, as shown in Fig. 12, C1 from the March dataset is similar in characteristics with C3 of the April dataset, which in turn is similar to C6 from the June dataset. This indicates that each country has shown different responses to contain the spread of COVID-19.

## Conclusion

In this paper, we present an innovative way to add explainability to the popular and commonly used SIR models for understanding countries’ responses to COVID-19 containment. The proposed tensor-based factorization approach enhances the interpretation of a detailed Bayesian analysis of a stochastic epidemiological model. By representing the 158 country-specific posterior distributions as a tensor and applying NTF to cluster the countries, we provide insights to learn the characteristics of countries and their movements as clusters in different time-periods. While SIR learns latent epidemiological parameters of different countries, NTF leverages SIR by representing latent epidemiological parameters in a tensor model to reveal latent features of countries. The experimental results are set in a meaningful way to gain many significant insights into the COVID-19 spread and the behaviour of the global responses of each country. Our observation on the results is that quick restrictions and controlling the transmission are the key to containing the spread of COVID-19 cases and avoiding future waves. The proposed work is generic, and hence can be extended to do similar things to assess the uptake and roll-out of vaccines. Moreover, this could help as a tool for understanding future pandemics. Another impactful direction of this research can be to include economic factors in the epidemiological model.

## Footnotes

1 For notional convenience, the recovery state *R* in SIR model is denoted as *Ru*.

2 Notations used in this section may not relate to the notations used in the previous section.

3 We use ISO-3166 alpha3 codes to represent country names.
